# Peripheral neuropathy predicts nuclear gene defect in patients with mitochondrial ophthalmoplegia

**DOI:** 10.1093/brain/awu279

**Published:** 2014-10-04

**Authors:** Alejandro Horga, Robert D. S. Pitceathly, Julian C. Blake, Catherine E. Woodward, Pedro Zapater, Carl Fratter, Ese E. Mudanohwo, Gordon T. Plant, Henry Houlden, Mary G. Sweeney, Michael G. Hanna, Mary M. Reilly

**Affiliations:** 1 MRC Centre for Neuromuscular Diseases, UCL Institute of Neurology and National Hospital for Neurology and Neurosurgery, Queen Square, London, WC1N 3BG, UK; 2 Department of Clinical Neurophysiology, Norfolk and Norwich University Hospital, Norwich, NR4 7UY, UK; 3 Neurogenetics Unit, National Hospital for Neurology and Neurosurgery, Queen Square, London, WC1N 3BG, UK; 4 Clinical Pharmacology Section, Hospital General Universitario, Alicante, 03010, Spain; 5 Oxford Medical Genetics Laboratories, Oxford University Hospitals NHS Trust, Oxford, OX3 7LE, UK; 6 National Hospital for Neurology and Neurosurgery, Queen Square, London, WC1N 3BG, UK

**Keywords:** mitochondrial DNA, mitochondrial DNA deletion, peripheral neuropathy, *POLG*, progressive external ophthalmoplegia

## Abstract

Progressive external ophthalmoplegia is a common clinical feature in mitochondrial disease caused by nuclear DNA defects and single, large-scale mitochondrial DNA deletions and is less frequently associated with point mutations of mitochondrial DNA. Peripheral neuropathy is also a frequent manifestation of mitochondrial disease, although its prevalence and characteristics varies considerably among the different syndromes and genetic aetiologies. Based on clinical observations, we systematically investigated whether the presence of peripheral neuropathy could predict the underlying genetic defect in patients with progressive external ophthalmoplegia. We analysed detailed demographic, clinical and neurophysiological data from 116 patients with genetically-defined mitochondrial disease and progressive external ophthalmoplegia. Seventy-eight patients (67%) had a single mitochondrial DNA deletion, 12 (10%) had a point mutation of mitochondrial DNA and 26 (22%) had mutations in either *POLG*, *C10orf2* or *RRM2B*, or had multiple mitochondrial DNA deletions in muscle without an identified nuclear gene defect. Seventy-seven patients had neurophysiological studies; of these, 16 patients (21%) had a large-fibre peripheral neuropathy. The prevalence of peripheral neuropathy was significantly lower in patients with a single mitochondrial DNA deletion (2%) as compared to those with a point mutation of mitochondrial DNA or with a nuclear DNA defect (44% and 52%, respectively; *P < *0.001). Univariate analyses revealed significant differences in the distribution of other clinical features between genotypes, including age at disease onset, gender, family history, progressive external ophthalmoplegia at clinical presentation, hearing loss, pigmentary retinopathy and extrapyramidal features. However, binomial logistic regression analysis identified peripheral neuropathy as the only independent predictor associated with a nuclear DNA defect (*P = *0.002; odds ratio 8.43, 95% confidence interval 2.24–31.76). Multinomial logistic regression analysis identified peripheral neuropathy, family history and hearing loss as significant predictors of the genotype, and the same three variables showed the highest performance in genotype classification in a decision tree analysis. Of these variables, peripheral neuropathy had the highest specificity (91%), negative predictive value (83%) and positive likelihood ratio (5.87) for the diagnosis of a nuclear DNA defect. These results indicate that peripheral neuropathy is a rare finding in patients with single mitochondrial DNA deletions but that it is highly predictive of an underlying nuclear DNA defect. This observation may facilitate the development of diagnostic algorithms. We suggest that nuclear gene testing may enable a more rapid diagnosis and avoid muscle biopsy in patients with progressive external ophthalmoplegia and peripheral neuropathy.

## Abstract (precis)

Mitochondrial ophthalmoplegia is a genetically heterogeneous disorder. Horga *et al.* investigate whether peripheral neuropathy can predict the underlying genetic defect in patients with progressive external ophthalmoplegia. Results indicate that neuropathy is highly predictive of a nuclear DNA defect and that it is rarely associated with single mitochondrial DNA deletions.

## Introduction

Progressive external ophthalmoplegia (PEO) is a common presentation and frequently the defining clinical feature of mitochondrial respiratory-chain disease. It is characterized by slowly progressive, painless, bilateral ptosis and generalized reduction of ocular movements in all directions of gaze that is not usually associated with diplopia or significant fluctuations (Lee and Brazis, 2002). Patients may develop additional muscular, neurological or systemic features, leading to a variety of syndromes that range from isolated chronic PEO to those with multisystem involvement such as Kearns-Sayre syndrome or mitochondrial neurogastrointestinal encephalomyopathy (Chinnery and Shoubridge, 2010). These must be distinguished from other non-mitochondrial disorders causing ptosis and ophthalmoplegia such as myasthenia gravis, oculopharyngeal muscular dystrophy, oculopharyngodistal myopathy or *MYH2*-related myopathy.

From a genetic perspective, PEO is associated with both primary and secondary mitochondrial DNA defects (Hirano and DiMauro, 2001). The former include single, large-scale mitochondrial DNA deletions, which are usually sporadic in occurrence and have a low transmission risk, and maternally-inherited mutations in mitochondrial tRNA or protein-coding genes (e.g. *MT-TL1, MT-TI* and *MT-ND4*) (Sweeney *et al.*, 1993; Raffelsberger *et al.*, 2001; Pulkes *et al.*, 2003; Smits *et al.*, 2007; Nesbitt *et al.*, 2013). The second group include inherited defects in nuclear-encoded genes involved in mitochondrial DNA replication and maintenance (e.g. *POLG*, *POLG2*, *C10orf2*, *SLC25A4*, *OPA1*, *SPG7*, *TYMP*, *RRM2B*, *TK2* and *DGUOK*) that cause multiple deletions and/or depletion of mitochondrial DNA (Agostino *et al.*, 2003; Hudson *et al.*, 2006, 2008; Fratter *et al.*, 2011; Garone *et al.*, 2011; Park *et al.*, 2011; Young *et al.*, 2011; Ronchi *et al.*, 2012; Tyynismaa *et al.*, 2012; Pfeffer *et al.*, 2014). PEO is indeed a clinical hallmark of patients with single mitochondrial DNA deletions but also the most common presenting feature in adults with nuclear DNA defects. Point mutations of mitochondrial DNA account for a smaller proportion of cases. For instance, PEO has been reported to occur in 12% of patients with the most frequent point mutation, the m.3243A>G transition in *MT-TL1* [tRNA^Leu(UUA/G)^] (Nesbitt *et al.*, 2013).

Peripheral neuropathy is also a well-recognized manifestation of mitochondrial disease, although its prevalence and characteristics vary considerably among the different syndromes and genetic causes. It is a major or a common feature of a variety of nuclear DNA defects (e.g. *TYMP-*, *MPV17*- and *POLG*-related disorders) but also of certain point mutations of mitochondrial DNA (e.g. m.8993T>G/C and m.3243A>G) (Holt *et al.*, 1990; Karadimas *et al.*, 2006; Kaufmann *et al.*, 2006; Garone *et al.*, 2011; Lax *et al.*, 2012). However, there are only anecdotal reports of peripheral neuropathy in patients with single mitochondrial DNA deletions (Eymard *et al.*, 1991; Reichmann *et al.*, 1991; Molnar *et al.*, 1996). This observation suggests that peripheral nerve involvement is extremely rare in this group of patients and contrasts with the widespread tissue distribution of deleted mitochondrial DNA species in some of them (e.g. Kearns-Sayre syndrome) (Moraes *et al.*, 1989; Ponzetto *et al.*, 1990; Brockington *et al.*, 1995).

Mitochondrial respiratory-chain diseases often present with a diverse constellation of clinical manifestations that may hinder the distinction between phenotypes. Certain combinations of symptoms, however, can suggest the specific aetiology. Based on initial clinical and genetic observations, we hypothesized that the presence of peripheral neuropathy might predict a nuclear DNA defect in patients with PEO.

The main objectives of the present study were to determine the frequency of peripheral neuropathy in a large cohort of patients with genetically-defined mitochondrial disease and PEO and to evaluate whether the presence of peripheral neuropathy or other clinical features could predict the underlying genetic defect in the same population. Secondary objectives were to define the proportion of genotypes among patients with PEO and to describe the characteristics of the peripheral neuropathy.

## Materials and methods

### Study design and patient selection

**Figure 1:**
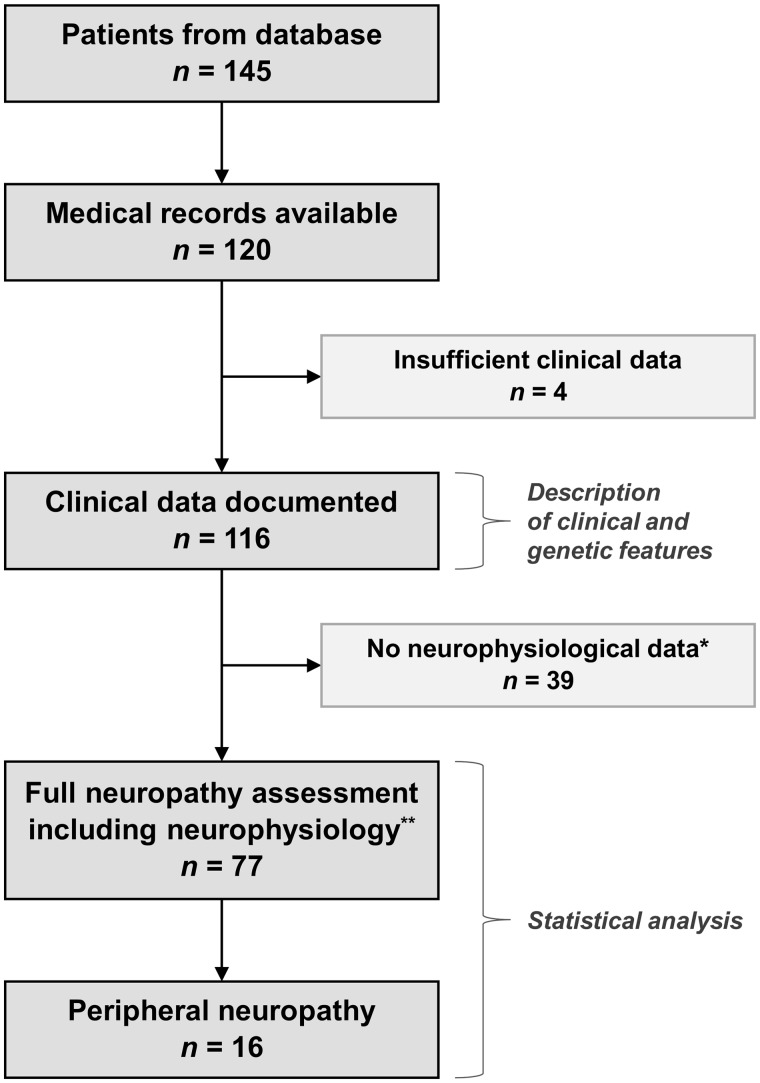
Flow diagram of the study. *Two patients had symptoms and/or signs suggestive of peripheral neuropathy but no documented neurophysiological study. **Neurophysiological studies of the lower ± upper limbs.

This was a retrospective, observational, single-centre study based on chart review of patients fulfilling the following inclusion criteria: diagnosis of PEO (progressive ptosis and restriction of extraocular motility in all directions of gaze) as judged by the examining neurologist; and confirmed genetic defect of either mitochondrial DNA or nuclear-encoded genes involved in mitochondrial DNA translation, replication or maintenance. Cases were identified from a database of all patients with mitochondrial disease and unaffected relatives assessed at the MRC Centre for Neuromuscular Diseases, National Hospital for Neurology and Neurosurgery, London, between 1985 and 2013 (Fig. 1). From a total of 145 candidates, medical records were available for review for 120 probands. Four patients were excluded due to insufficient clinical information. Demographic, clinical and paraclinical data from the resulting 116 patients were abstracted into a standardized form.

This study was performed under the ethical guidelines issued by our institution for clinical audit studies. Written informed consent was obtained from all subjects before genetic testing.

### Data collection

The following features were systematically collected: gender; date of birth; family history of relatives with similar symptoms or phenotype; ptosis; ophthalmoparesis; pigmentary retinopathy on fundoscopic examination; hearing loss, symptomatic or confirmed on audiogram; dysarthria; dysphagia; exercise intolerance; limb muscle weakness; large-fibre peripheral neuropathy; pyramidal signs; ataxia, cerebellar or sensory; seizures or epilepsy; stroke, stroke-like episodes or territorial infarct on neuroimaging; movement disorders, including myoclonus, dystonia and parkinsonism; diabetes; cardiac disorders; and date of last follow-up or death. Other clinical characteristics were also recorded if considered relevant. Complete medical assessments were assumed and a feature was considered as absent when the history or examination was explicitly reported as being unremarkable or normal except for cardiac disorders, which were considered missing if not reported. Emphasis was placed on reviewing and collecting symptoms potentially related to peripheral neuropathy and findings from the motor and sensory examination. Although subject to recall bias, an approximate date (year) of symptoms onset and the presenting feature were also recorded. Muscle biopsy findings and laboratory tests, including creatine kinase, lactate, lactate/pyruvate ratio, renal and thyroid function tests, glucose, HbA1c (glycated haemoglobin), folate and vitamin B12 levels, were collected where available.

Neurophysiology reports were available for review in 90 cases. In 13 of them, nerve conduction studies had been performed only in the upper limbs. Of the remaining 77 patients, 43 had at least one sensory and motor nerve studied in one upper and one lower limb; 24 had less than one sensory and motor nerve studied in one upper and one lower limb; and 10 had nerve conduction studies performed only in the lower limbs. Of these 77 patients, 74 had nerve conduction studies with or without EMG done using standard methods at the National Hospital for Neurology and Neurosurgery, UK. In three cases, neurophysiological studies were performed at other tertiary hospitals. Two authors (A.H. and J.C.B.) reviewed the reports for technical and interpretation accuracy. Results were compared to normative data on healthy individuals.

Molecular genetic analysis of mitochondrial DNA extracted from blood, muscle or urine had been performed at the Neurogenetics Unit, National Hospital for Neurology and Neurosurgery, London. The presence of point mutations of mitochondrial DNA was confirmed by PCR-restriction fragment length polymorphism (m.3243A>G), sequence analysis of *MT-TL1* (m.3260A>G) (Sweeney *et al.*, 1993), or sequence analysis of the entire mitochondrial DNA and mismatch PCR (m.12294G>A and m.11232T>C) (Pulkes *et al.*, 2003). Large-scale mitochondrial DNA rearrangements were confirmed by long-range PCR and Southern blotting. Molecular analysis of nuclear genes extracted from blood or muscle had been performed by direct sequencing at the Oxford Medical Genetics Laboratories.

### Patient classification

For descriptive purposes, patients were classified into four phenotype groups: chronic PEO with or without bulbar symptoms (dysarthria or dysphagia), limb weakness or other features but no CNS involvement; chronic PEO with CNS involvement, including pigmentary retinopathy and hearing loss (chronic PEO/CN); Kearns-Sayre syndrome, as defined by the triad PEO, pigmentary retinopathy and onset before age 20 years, plus at least one of the following: cerebellar ataxia, cardiac conduction block and CSF protein >100 mg/dl; and sensory ataxia, neuropathy, dysarthria and ophthalmoplegia (SANDO), as defined by the combination of sensory ataxia, neuropathy, dysarthria and ophthalmoplegia. For analytical purposes, patients were grouped into three genotype groups: point mutations of mitochondrial DNA; single mitochondrial DNA deletions; and nuclear DNA defects, including multiple mitochondrial DNA deletions confirmed in muscle without an identified nuclear gene defect.

### Data analysis

First we performed a descriptive analysis of the clinical and genetic features of all patients included in the study (*n = *116). Second, in the subgroup of patients with neurophysiological evaluation of the lower ± upper limbs only (*n = *77), we performed the following analyses: (i) univariate and multivariate analyses with nuclear DNA defect versus mitochondrial DNA defect as a binary or ternary outcome to evaluate the associations with predictor variables; (ii) decision tree analysis to investigate individual variables with optimal performance in genotype classification; and (iii) assessment of the diagnostic efficiency of the individual predictors.

Categorical variables were presented as number and percentage. Contingency tables were analysed using the Pearson’s *χ*^2^ test or the Fisher’s exact test, phi (φ) coefficients, and odds ratios (ORs) with their corresponding 95% confidence intervals (CIs) where appropriate. The Shapiro-Wilk test was used for normality testing. Non-parametric data were presented as median and range or interquartile range (IQR) and compared using the Mann-Whitney U test or the Kruskal–Wallis test.

Univariate analyses were performed with the Pearson’s χ^2^ test or the Fisher’s exact test for categorical variables and with binomial logistic regression for continuous variables. Forced entry logistic regression was used to examine potential confounders and interactions. Multivariate analyses were conducted using binomial and multinomial logistic regression with forward stepwise and forced entry procedures, respectively. The selection and number of variables to be included in the analyses were guided by *P*-values on univariate testing (<0.05) and number of observations per independent variable (≥10). The multinomial logistic regression model with the best fit was selected using the Akaike’s Information Criterion.

Decision tree analysis is a statistical method that can be used to create a tree-based classification of cases into groups based on a set of independent variables, with the purpose of identifying a useful subset of variables that allow distinction between groups. The same variables entered in the multinomial logistic regression analysis were used to construct a decision tree based on the exhaustive ‘Chi-squared automatic interaction detection’ (CHAID) algorithm with the following adjustments: ≥10 and ≥5 cases per parent node and child node, respectively; automatic maximum tree depth; Pearson’s χ^2^ statistic; significant level for splitting nodes set at <0.05 with Bonferroni correction; and 10-fold cross-validation.

The statistical analyses were performed using IBM SPSS Statistics version 21 (IBM). All tests were two-tailed and *P*-values <0.05 were considered statistically significant. A Bonferroni correction was applied by multiplying *P*-values by the number of comparisons where appropriate. The efficiency of each predictor for the diagnosis of a nuclear DNA defect was examined with its sensitivity, specificity, positive and negative predictive values and likelihood ratios, calculated with MedCalc version 12 (http://www.medcalc.org).

## Results

### Sample characteristics

Sixty females and 56 males from 116 apparently unrelated pedigrees were included in the study (full genetic and clinical features in Supplementary Table 1). The median age at disease onset was 21.3 years (*n = *111, IQR 13.6–34.7). PEO was the predominant presenting symptom in 90 patients (78%). Other patients presented with the following clinical manifestations or a combination of them: developmental delay, growth retardation, exercise intolerance, limb weakness, abnormal sensation, gait disturbance, visual loss, hearing loss, parkinsonism, seizures, stroke-like episodes, diabetes and heart block. The median follow-up time from disease onset was 22.3 years (*n = *111, IQR 11.0–32.9).

All patients had both ptosis and external ophthalmoplegia with the exception of one patient with severe ophthalmoplegia and no ptosis, and one patient with severe ptosis and abnormal ocular movements but no ophthalmoplegia. Phenotype frequencies were as follows: 58 patients (50%) had chronic PEO; 41 (35%) had chronic PEO/CN, including one patient with mitochondrial encephalopathy, lactic acidosis and stoke-like episodes (MELAS)/chronic PEO and three with maternally inherited diabetes and deafness/chronic PEO overlap syndromes; 11 (10%) patients had Kearns-Sayre syndrome; and six (5%) had SANDO. The clinical features for each phenotype are summarized in Supplementary Table 2.

**Figure 2:**
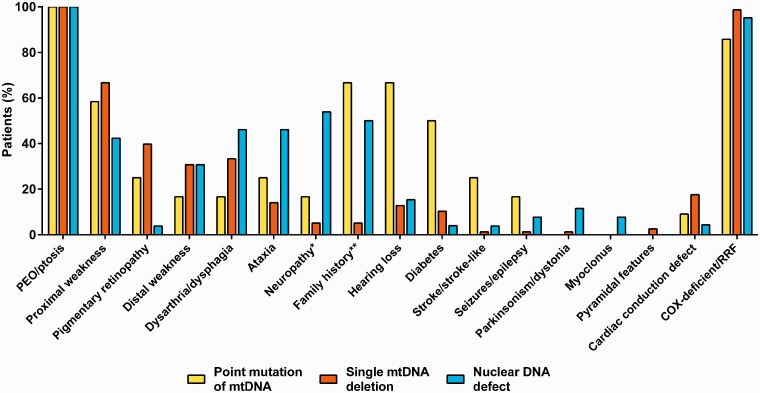
Frequency of clinical features of patients according to the genotype (*n = *116). Forward slash indicates ‘and/or’. *Symptoms and/or signs suggestive of peripheral neuropathy. **Family history of relatives with similar symptoms or phenotype. COX-deficient/RRF = cytochrome c oxidase-deficient fibres and/or ragged-red fibres in muscle histochemistry.

Ninety patients (78%) had a primary mitochondrial DNA defect. Of them, 78 (67%) had a single deletion and 12 (10%) had a transition mutation in *MT-TL1*, *MT-TL2* or *MT-ND4*. Eighteen patients (16%) had pathogenic variants in either *POLG*, *C10orf2* or *RRM2B*, and eight (7%) had multiple mitochondrial DNA deletions detected in muscle without an identified nuclear gene defect. Of these eight patients, seven had undergone sequence analysis of the coding region of *POLG* and a targeted mutation screen of *C10orf2*, and five had also undergone analysis of the coding region of *RRM2B* and *SLC25A4*. The clinical features for each genotype are shown in Fig. 2 and Supplementary Table 2.

**Figure 3:**
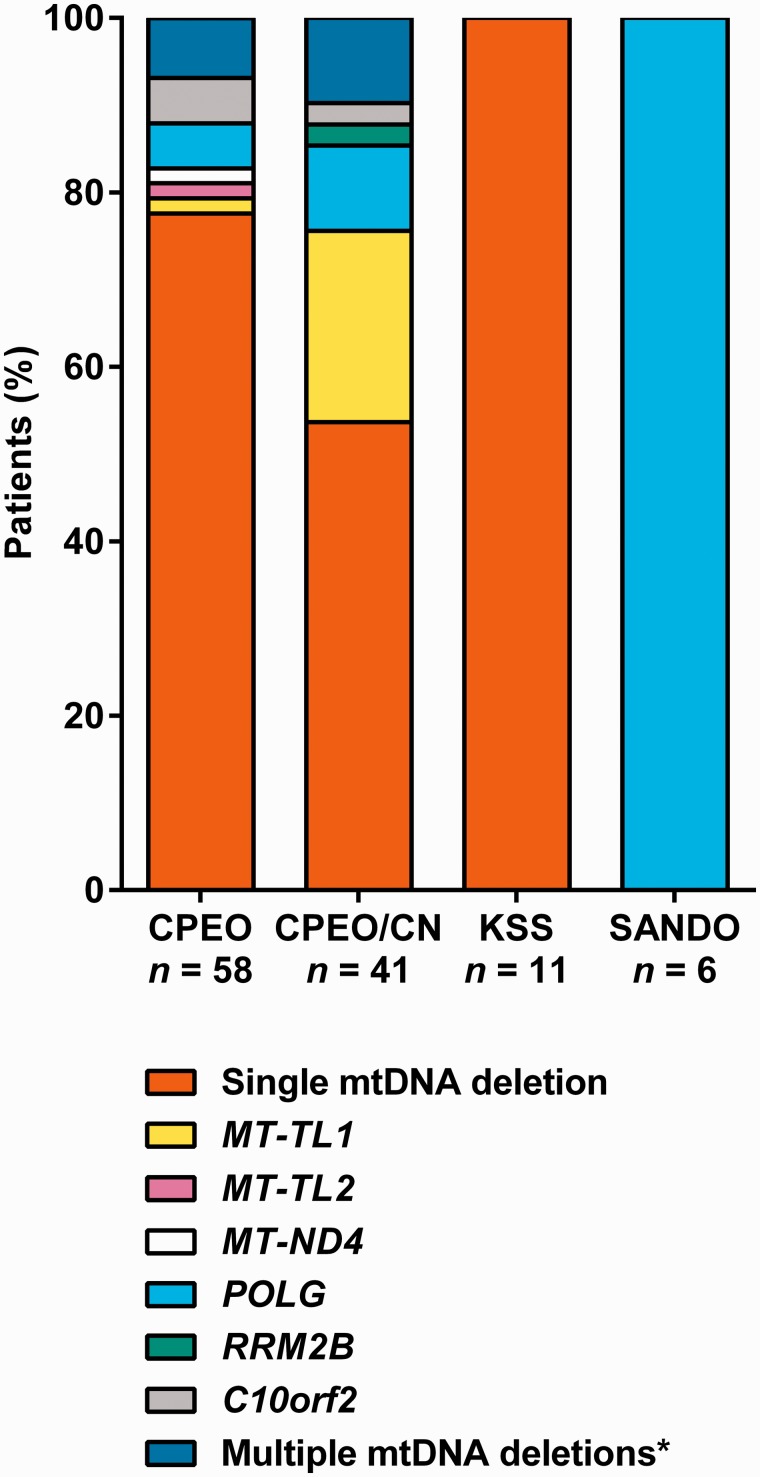
Phenotype-genotype distribution of patients (*n* = 116): proportion of patients for each phenotype with single mitochondrial DNA deletions, point mutations of mitochondrial DNA (*MT-TL1*, *MT-TL2* and *MT-ND4* genes), mutations in nuclear genes (*POLG*, *C10orf2* and *RRM2B*) or multiple mitochondrial DNA deletions in muscle without an identified nuclear gene defect (*). CPEO = chronic PEO. CPEO/CN = chronic PEO with CNS involvement. KSS = Kearns-Sayre syndrome.

There was a significant difference in the overall distribution of phenotypes between genotypes (*P < *0.001) (Fig. 3 and Supplementary Table 3). Subgroups analysis disclosed direct associations (positive φ coefficient) between chronic PEO/CN and point mutations of mitochondrial DNA (*P = *0.004), between SANDO and nuclear DNA defects (*P < *0.001), and between Kearns-Sayre syndrome and single mitochondrial DNA deletions (*P = *0.015). Only the first two associations, however, retained statistical significance after Bonferroni adjustment (*P = *0.048, *P < *0.001 and *P = *0.132, respectively).

From the total of 116 patients included in the study, 77 (66%) had neurophysiological studies performed in the lower ± upper limbs. The median age at examination was 46.9 years (IQR 30.3–54.4). Except for gender distribution, frequency of proximal muscle weakness and frequency of peripheral neuropathy symptoms/signs, there were no demographic and clinical differences between this group of 77 patients and the remaining 39 (Supplementary Table 4). Of this latter group, one had signs and symptoms suggestive of sensory neuropathy (chronic PEO/CN phenotype; *C10orf2* mutation), and one had reduced vibration sense at the malleolus and absent right ankle jerk at age 73 years (chronic PEO phenotype; single mitochondrial DNA deletion).

Of the 77 patients with neurophysiological studies performed in the lower ± upper limbs, 16 (21%) had a large-fibre peripheral neuropathy confirmed by nerve conduction studies: 13 patients with and three patients without sensory or motor symptoms and/or signs. Five patients had symptoms and/or signs but no neuropathy on nerve conduction studies: two asymptomatic with abnormal sensory examination; two with symptoms but normal examination; and one with sensory symptoms and reduced ankle jerks concomitant to gold salt therapy for rheumatoid arthritis. In this latter case, nerve conduction studies showed reduced sural sensory nerve action potential amplitudes (4 and 3 μV) that remained stable or improved over time (5 and 8 μV). One patient had a severe L4/5 radiculopathy with asymmetric sensory nerve action potential amplitudes in the lower limbs. The remaining 55 patients had no symptoms and no neurophysiological evidence of peripheral neuropathy (sural sensory nerve action potential amplitude: median 17 μV; range 7–50 μV).

### Peripheral neuropathy

The genetic and clinical features of the 16 patients with peripheral neuropathy are summarized in Table 1 (neurophysiological data in Supplementary Table 5). In this group, the median age at disease onset was 31.9 years (IQR 24.1–49.7). One patient (6%) had a single mitochondrial DNA deletion, four (25%) had the m.3243A>G mutation, nine (56%) had *POLG* mutations, one (6%) had multiple mitochondrial DNA deletions in muscle with negative sequence analysis of *POLG* and *C10orf2*, and one (6%) had multiple mitochondrial DNA deletions in muscle with negative sequence analysis of *POLG*, *C10orf2*, *RRM2B* and *SLC25A4*. Except for diabetes in three patients with m.3243A>G, none of them had endocrine–metabolic disorders that could account for the peripheral neuropathy.

**Table 1:** Genetic and clinical features of patients with peripheral neuropathy confirmed by neurophysiological examination

ID	Gender	Gene	Mutation	Multiple mtDNA deletions	Muscle histochemistry	Age at onset (years)	Main clinical features	Peripheral neuropathy				
								Sensory symptoms	Sensory signs	Length- dependent^f^	Sensory / motor	Axonal / other
017^a^	M		Single mtDNA deletion		COX−, RRF	43	PEO, ptosis, dysphagia, mild proximal weakness, exercise intolerance	−	−	+	Sensory	n/a
065^a^	M	*MT-TL1*	m.3243A>G		RRF	57	PEO, ptosis, proximal weakness, hearing loss, hemispheric stroke, epilepsy	−	−	+	Sensory	n/a
124	M	*MT-TL1*	m.3243A>G		n/a	31	PEO, ptosis, hearing loss, stroke-like episode with seizures, lactic acidosis, diabetes	−	−	+	Sensory > motor	Mixed
128	F	*MT-TL1*	m.3243A>G		n/a	50	PEO, ptosis, maculopathy, hearing loss, mild ataxia, diabetes	−	+	+	Sensory > motor	Mixed
129	M	*MT-TL1*	m.3243A>G		n/a	31	PEO, ptosis, proximal weakness, hearing loss, retinopathy/maculopathy, diabetes, LVH	−	+	+	Sensory + motor	Mixed
094	F	*POLG*	p.A467T; p.A467T	n/a	COX−, RRF	23	PEO, ptosis, dysarthria, proximal and distal weakness, ataxia, hemispheric and cerebellar stroke, epilepsy	−	+	+	Sensory + motor	Mixed
093	M	*POLG*	p.A467T; p.A467T	+	COX−, RRF	14	PEO, ptosis, dysarthria, dysphagia, ataxia, vestibular dysfunction	+	+	n/a	Sensory	Unexcitable
016	M	*POLG*	p.A467T; p.W748S	n/a	n/a	30	PEO, ptosis, dysarthria, ataxia, parkinsonism (abnormal DAT scan)	+	+	n/a	Sensory	Unexcitable
033	M	*POLG*	p.A467T; p.W748S	+	COX−, RRF	43	PEO, ptosis, mild dysarthria, ataxia, cognitive dysfunction	+	+	n/a	Sensory	Unexcitable
023	M	*POLG*	p.A467T; p.L559P^b^	n/a	n/a	63	PEO, hearing loss, parkinsonism (abnormal DAT scan)	+	+	−	Sensory > motor	Axonal
096	M	*POLG*	p.A467T; p.*1240Q	+	COX−, RRF	18	PEO, ptosis, dysarthria, ataxia, myoclonus, dystonia	+	+	−	Sensory > motor	Axonal
095	M	*POLG*	p.P587L;^c^ p.R1081dup^d^	+	COX−, RRF	16	PEO, ptosis	−	+	+	Sensory + motor	Axonal
098^a^	M	*POLG*	p.P587L; p.R227W	+	COX−, RRF	54	PEO, ptosis, dysarthria, dysphagia, proximal and distal weakness, ataxia, cachexia	+	+	n/a	Sensory > motor	Axonal
122	M	*POLG*	p.Y955C	+	COX−, RRF	48	PEO, ptosis, dysarthria, tremor, ataxia, vestibular dysfunction	−	+	+	Sensory	Axonal
118	M	Unknown		+^e^	n/a	33	PEO, ptosis, proximal weakness, ataxia, vestibular dysfunction	−	+	−	Sensory > motor	Axonal
019	M	Unknown		+	COX−, RRF	27	PEO, ptosis, hearing loss, distal weakness, myoclonus, epilepsy	+	+	+	Sensory > motor	Axonal

^a^Probable neuropathy: limited neurophysiological data available.

^b^Probably pathogenic mutation: not reported in the Human POLG Mutation Database (tools.niehs.nih.gov/polg).

^c^Also heterozygous for the p.T251I variant usually found in *cis* with p.P587L.

^d^Previously reported as a probable recessive mutation.

^e^Multiple mitochondrial DNA deletions also confirmed in muscle from affected sibling.

^f^Sensory component.

F = female; M = male; COX− = cytochrome *c* oxidase deficient fibres; DAT = dopamine transporter; LVH = left ventricular hypertrophy; mixed = mixed axonal and mild conduction slowing; RRF = ragged-red fibres; + = present; − = absent; n/a = not available/insufficient data.

In all the patients with a primary mitochondrial DNA defect, the peripheral neuropathy was asymptomatic. The neurophysiological pattern was consistent with a length-dependent, predominantly sensory or sensorimotor neuropathy. In two patients, the data available was insufficient to characterize the neuropathy further. In three patients with m.3243A>G and diabetes, the peripheral neuropathy was axonal with mild slowing of sensory and motor conduction velocities. Only one patient with absent sensory and motor responses in the lower limbs had distal neurogenic changes on EMG.

The presenting manifestation in patients with a nuclear DNA defect and peripheral neuropathy was a variable combination of upper or lower limb sensory symptoms and ataxia with or without other features (PEO, dysarthria, parkinsonism or myoclonic jerks) in seven patients; PEO in two patients; stroke and epilepsy in one patient; and epilepsy and myoclonic jerks in one patient. All of them had abnormal sensory examination and abnormal tendon reflexes, and three had distal limb weakness.

The neurophysiological patterns in patients with a *POLG* mutation were as follows: unobtainable sensory responses and normal motor studies in three patients; non-length dependent, predominantly sensory, axonal neuropathy with distal active and chronic neurogenic changes on EMG in two patients; and length-dependent, sensory or sensorimotor axonal neuropathy in three patients, one of them with mild slowing of sensory and motor conduction velocities. In one patient, data was incomplete but suggestive of a predominantly sensory axonal neuropathy. The neurophysiological pattern in patients with multiple mitochondrial DNA deletions without an identified nuclear gene defect was consistent with a non-length dependent, predominantly sensory neuropathy with distal and proximal chronic neurogenic changes on EMG in one case, and with a length-dependent, predominantly sensory axonal neuropathy in another case.

### Predictors for nuclear DNA defect

The demographic and clinical characteristics of patients with neurophysiological studies of the lower ± upper limbs classified by genotype are summarized in Supplementary Tables 6 and 7. Age at disease onset, gender, family history, PEO/ptosis as the presenting feature, pigmentary retinopathy, peripheral neuropathy and parkinsonism/dystonia were significantly different in patients with a nuclear DNA defect as compared to patients with a primary mitochondrial DNA defect (point mutation or single deletion) evaluated as a group. The presence of ataxia was also associated with a nuclear DNA defect; after adjusting for the presence of peripheral neuropathy, however, this parameter did not retain statistical significance and therefore was not entered in the multivariate analysis. Differences were found in the distribution of the following characteristics between the three individual genotypes: age at disease onset, gender, family history, PEO/ptosis as the presenting feature, pigmentary retinopathy, hearing loss, ataxia, seizures/epilepsy, stroke/stroke-like episodes, parkinsonism/dystonia, peripheral neuropathy and diabetes.

Logistic regression analyses were performed to determine the independent factors associated with a nuclear DNA defect as a binary or ternary outcome (Tables 2 and 3). Binomial logistic regression identified peripheral neuropathy as the only independent predictor associated with nuclear DNA defect (*P = *0.002; OR 8.43, 95% CI 2.24–31.76). Multinomial logistic regression was conducted using nuclear DNA defect as the reference group. Three variables were identified as significant predictors of the genotype: peripheral neuropathy, family history and hearing loss. The absence of peripheral neuropathy and a negative family history were significant in differentiating patients with a single mitochondrial DNA deletion from those with a nuclear DNA defect (*P < *0.001; OR 55.90, 95% CI 5.96–524.12; and *P = *0.005; OR 9.35, 95% 1.95–44.82, respectively). The absence of hearing loss was significant in differentiating patients with a nuclear DNA defect from those with a point mutation of mitochondrial DNA (*P = *0.007; OR 0.04, 95% CI 0.004–0.43). Diagnostic properties of the three predictor variables are summarized in Table 4.

**Table 2:** Binomial logistic regression analysis of patients classified by genotype (nuclear DNA defect versus mitochondrial DNA defect; *n* = 77)

	B (SE)	*P*	OR	95% CI
Peripheral neuropathy (present)	2.13 (0.68)	0.002	8.43	2.24–31.76
Gender (male)	1.43 (0.73)	0.051	4.16	0.99–17.41

Model χ^2^ (2) = 21.0, *P* < 0.001; Hosmer and Lemeshow *P* = 0.931; R^2^ (Nagelkerke) = 0.356; overall accuracy of classification = 81.3%. SE = standard error.

**Table 3:** Multinomial logistic regression analysis of patients classified by genotype (*n* = 77)

	Nuclear DNA defect versus single mitochondrial DNA deletion			Nuclear DNA defect versus point mutation of mitochondrial DNA		
	OR (95% CI)	B (SE)	*P*	OR (95% CI)	B (SE)	*P*
**Peripheral neuropathy**						
Present (ref.)	1			1		
Absent	55.90 (5.96–524.12)	4.02 (1.14)	0.000	3.13 (0.34–28.42)	1.14 (1.13)	0.312
**Family history**						
Positive (ref.)	1			1		
Absent	9.35 (1.95–44.82)	2.24 (0.80)	0.005	0.48 (0.06–3.92)	−0.73 (1.07)	0.494
**Hearing loss**						
Present (ref.)	1			1		
Absent	0.42 (0.04–3.98)	−0.88 (1.15)	0.447	0.04 (0.004–0.43)	−3.15 (1.17)	0.007

Reference category for equations = nuclear DNA defect; Model χ^2^ (6) = 54.6, *P* < 0.001; Goodness-of-fit (Pearson) *P* = 0.881; R^2^ (Nagelkerke) = 0.607; overall accuracy of classification = 80.5%. SE = standard error.

**Table 4:** Test characteristics of peripheral neuropathy, family history and hearing loss in the diagnosis of patients with a nuclear DNA defect (*n* = 77)

	Prevalence	Sensitivity	Specificity	PPV	NPV	+LR	−LR
**Peripheral neuropathy**	21%	52%	91%	69%	83%	5.87	0.52
**Positive family history**	26%	43%	80%	45%	79%	2.18	0.71
**Hearing loss**	21%	14%	77%	19%	70%	0.62	1.12

PPV = positive predictive value; NNV = negative predictive value; +LR = positive likelihood ratio; −LR = negative likelihood ratio.

### Decision tree

**Figure 4:**
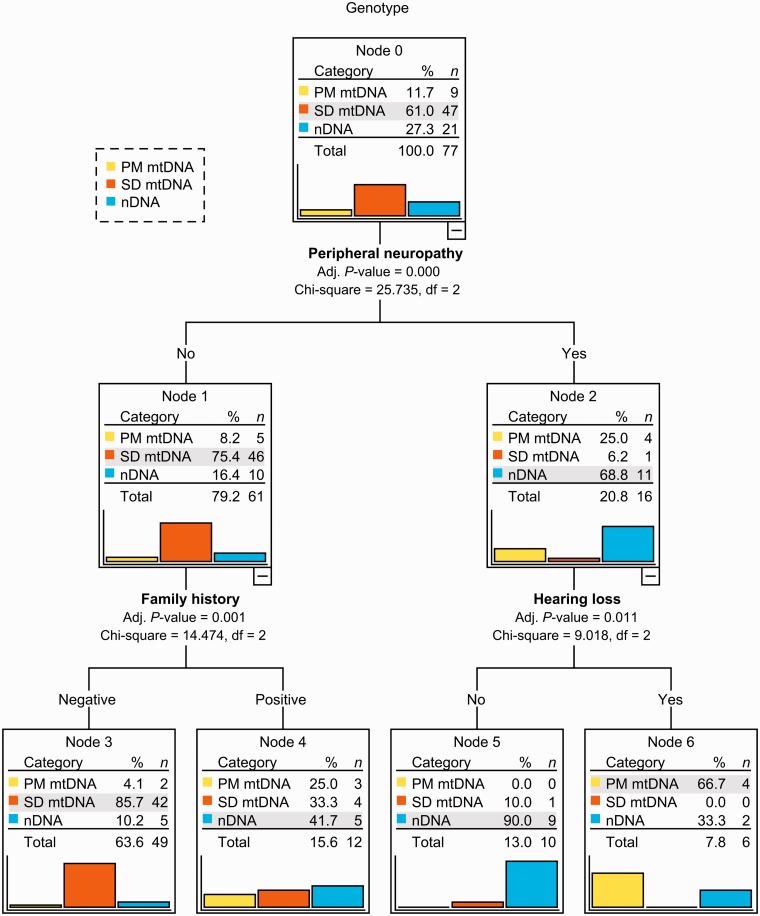
Ten-fold cross-validated, exhaustive Chi-squared automatic interaction decision tree for classification of genotypes according to individual clinical features (*n = *77). Percentages and bars for each genotype (category) indicate the relative proportion of patients within each node. Total percentages represent the proportion of patients in each node relative to the initial sample. Adj. *P*-value = adjusted *P*-value; df = degrees of freedom; nDNA = nuclear DNA defect; PM mtDNA = point mutation of mitochondrial DNA; SD mtDNA = single mitochondrial DNA deletion.

A decision tree was constructed using the following variables: gender, family history, PEO/ptosis as the presenting feature, pigmentary retinopathy, hearing loss and peripheral neuropathy (Fig. 4). Age at disease onset was converted into the dichotomous variable ‘onset before age 30 years’ and also entered in the analysis. Based on χ^2^ statistics, the variables with highest discriminatory power were peripheral neuropathy [χ^2^(2) = 25.7, *P < *0.001], which split the parent node into two child nodes (nodes 1 and 2), followed by family history [χ^2^(2) = 14.5, *P = *0.001] and hearing loss [χ^2^(2) = 9.0, *P = *0.011]), which split nodes 1 and 2 into four terminal nodes (3 to 6).

The highest probability (86%) of having a single mitochondrial DNA deletion was observed among patients with no peripheral neuropathy and a negative family history (node 3); the highest probability (90%) of having a nuclear DNA defect was observed among patients with peripheral neuropathy and no hearing loss (node 5); and the highest probability (67%) of having a point mutation of mitochondrial DNA was detected among patients with peripheral neuropathy and hearing loss (node 6). The overall classification accuracy of the decision tree was 78% (89%, 67% and 44% for single mitochondrial DNA deletion, point mutation of mitochondrial DNA and nuclear DNA defect, respectively).

## Discussion

The main findings of this study are: (i) the most common genetic defect associated with PEO in patients with mitochondrial disease is a single mitochondrial DNA deletion, in line with previous studies (Zeviani *et al.*, 1988; Holt *et al.*, 1989; Moraes *et al.*, 1989; Rodríguez-Hernández *et al.*, 2000; Jiménez Caballero *et al.*, 2007; Martikainen *et al.*, 2012); (ii) peripheral neuropathy is a rare clinical feature in patients with a single mitochondrial DNA deletion; and (iii) in the present patient sample, among several individual clinical features, peripheral neuropathy was the most important in predicting the genetic defect in patients with PEO caused by mitochondrial disease, followed by family history and hearing loss.

### Genotypes and clinical features

In this sample of 116 probands with genetically-defined mitochondrial disease and PEO, 67% of cases were due to a single mitochondrial DNA deletion. Fifty-seven per cent of these patients had a chronic PEO phenotype with bulbar or limb weakness. The remaining 43% had chronic PEO/CN or Kearns-Sayre syndrome. The median age of onset was 7.3 years lower in patients with chronic PEO/CN than in patients with chronic PEO, consistent with previous studies evaluating the natural history of patients with a single mitochondrial DNA deletion (Aure *et al.*, 2007). As expected from the phenotype classification, patients with Kearns-Sayre syndrome had also a younger age at disease onset. Except for pigmentary retinopathy and hearing loss, and ataxia and pyramidal features in patients with Kearns-Sayre syndrome, the frequency of other CNS symptoms such as epilepsy, myoclonus or extrapyramidal features was low. Four patients with a single mitochondrial DNA deletion were said to have a family history of similar symptoms or phenotype but this was not confirmed genetically.

Ten per cent of patients had a point mutation of mitochondrial DNA. Three had a chronic PEO phenotype, two of them with exercise intolerance and proximal muscle weakness; these have been described in detail elsewhere (Sweeney *et al.*, 1993; Pulkes *et al.*, 2003). Nine patients had the m.3243A>G mutation, all of them with a chronic PEO/CN phenotype. The most prevalent extramuscular manifestations in this group were hearing loss and diabetes. These were also the commonest features (51% and 42%, respectively) in a large cohort of 129 individuals with the m.3243A>G mutation (Nesbitt *et al.*, 2013). In only three cases, symptoms conformed to well-recognized clinical syndromes (maternally inherited diabetes and deafness and MELAS) in combination with PEO.

Sixteen per cent of patients had a nuclear DNA defect. Three nuclear genes were associated with PEO in the present study: *POLG*, *C10orf2* and *RRM2B*. Mutations in these genes were responsible for 11%, 3% and 1% of all cases, respectively. In line with previous studies, p.A467T followed by p.W748S were the most common variants identified in patients with two *POLG* mutations (Tang *et al.*, 2011; Neeve *et al.*, 2012). Six patients had SANDO: five of them were compound heterozygous for pathogenic *POLG* mutations and one was heterozygous for a pathogenic *POLG* mutation. One additional patient had a clinical picture suggestive of SANDO; in this case, however, the ataxia might have been secondary to a cerebellar infarct and was classified as chronic PEO/CN. One patient with the p.T423S variant in *C10orf2* had a phenotype suggestive of SANDO except for the absence of ophthalmoplegia. Other patients with *C10orf2* had chronic PEO with no CNS features.

Seven per cent of patients had multiple mitochondrial DNA deletions in muscle without an identified nuclear gene defect and had chronic PEO with or without CNS involvement. Except for three cases of isolated chronic PEO, no specific combination of symptoms was observed that was common to any two or more of these patients.

### Peripheral neuropathy

In this study, peripheral neuropathy was an extremely rare feature in patients with a single mitochondrial DNA deletion: only 1 of 47 patients (2%) had a probable subclinical sensory neuropathy. In contrast, the prevalence of peripheral neuropathy confirmed by nerve conduction studies in patients with point mutations of mitochondrial DNA and nuclear DNA defects was significantly higher (44% and 52%, respectively).

The mechanisms of single deletion formation remain incompletely understood, although the process probably takes place during oogenesis or early embryogenesis (Pitceathly *et al.*, 2012). Deleted genomes could subsequently populate different tissues and expand clonally during development. Disparities in tissue distribution and mutation load have been proposed to account for at least part of the phenotypic variability observed among patients with chronic PEO, chronic PEO/CN and Kearns-Sayre syndrome (Moraes *et al.*, 1989; Ponzetto *et al.*, 1990; Aure *et al.*, 2007). Deleted mitochondrial DNA species have indeed been demonstrated in most tissues in patients with Kearns-Sayre syndrome, in keeping with the multisystemic nature of the disease (Ponzetto *et al.*, 1990; Kageyama *et al.*, 1991; Brockington *et al.*, 1995; Boles *et al.*, 1998). Theoretically, the observed low frequency of neuropathy in patients with a single mitochondrial DNA deletion could be explained by differences in mutation load or tissue susceptibility. To our knowledge, however, the presence and load of single mitochondrial DNA deletions has not been studied in peripheral somatic nerves in patients with mitochondrial disease.

The prevalence of neuropathy in patients with m.3243A>G has been reported to range between 5% and 77% (Chinnery *et al.*, 1997; Kärppä *et al.*, 2003; Kaufmann *et al.*, 2006; Liu *et al.*, 2012). In a previous study, neurophysiological examination of seven patients with m.3243A>G disclosed a peripheral neuropathy with mixed axonal and demyelinating features in six cases and uniform demyelinating features in one case. Four of these patients had diabetes (Kärppä *et al.*, 2003). In another study, from a total of 23 patients with m.3243A>G and peripheral neuropathy, the neurophysiological pattern was axonal, mixed or demyelinating in 52%, 30% and 17% of cases, respectively. Nine of these patients had abnormal fasting glucose levels (Kaufmann *et al.*, 2006). We identified a total of nine patients with PEO and m.3243A>G, three of them with peripheral neuropathy and diabetes. Nerve conduction studies were consistent with an axonal neuropathy with mild slowing of conduction velocities, in a pattern reminiscent of that seen in individuals with distal symmetrical diabetic polyneuropathy without mitochondrial disease (Partanen *et al.*, 1995; Herrmann *et al.*, 2002). Although the presence of the m.3243A>G mutation has been demonstrated in peripheral nerves (Love *et al.*, 1993), whether the neuropathy in these patients is due to mitochondrial dysfunction, diabetes or both is unclear.

A sensory ataxic neuropathy or neuronopathy is a frequent feature in patients with recessive *POLG* mutations, particularly in adults, and often presents in association with dysarthria and PEO (Fadic *et al.*, 1997; Van Goethem *et al.*, 2003; Tang *et al.*, 2011; Lax *et al.*, 2012). Nerve conduction studies usually show absent sensory responses in the lower limbs and absent or reduced sensory nerve action potential amplitudes in the upper limbs, and motor axonal involvement is also described in some cases (Fadic *et al.*, 1997; Lax *et al.*, 2012). The existence of multiple deletions and depletion of mitochondrial DNA as well as mitochondrial respiratory-chain defects have been confirmed in dorsal root ganglia neurons from one patient with recessive *POLG* mutations, establishing a direct aetiological link between mitochondrial dysfunction and the neuronopathy (Lax *et al.*, 2012). In the present study, 69% of patients with PEO due to *POLG* mutations had peripheral neuropathy, a higher figure than previously reported (Horvath *et al.*, 2006), and *POLG* mutations were the commonest cause of peripheral neuropathy. Most patients presented with a predominantly sensory neuropathy and five of them had clinical and neurophysiological features consistent with a sensory neuronopathy: sensory deficits with or without ataxia, plus a non-length dependent sensory axonal neuropathy or unobtainable sensory responses.

Peripheral neuropathy has been reported in only a small number of patients with PEO and *C10orf2* or *RRM2B* mutations (Fratter *et al.*, 2010; Pitceathly *et al.*, 2012). We did not observe symptoms suggestive of neuropathy in patients with mutations in these genes except in one patient that developed a possible peripheral neuropathy related to gold salt therapy in the past. In this case, a follow-up study after a 7-year interval did not reveal progression of the neurophysiological abnormalities.

### Predictive factors

Among the individual clinical features with unequal distribution between genotype groups, including age of onset, gender, family history, PEO/ptosis as the presenting feature, pigmentary retinopathy and hearing loss, peripheral neuropathy was the one with highest ability to predict and discriminate between genotypes, particularly between nuclear DNA defect and single mitochondrial DNA deletion, as shown by both logistic regression and decision tree analysis. The odds of a patient with PEO and peripheral neuropathy having a nuclear DNA defect was 8.43 (95% CI 2.24–31.76) times higher than those of a patient with PEO but no peripheral neuropathy. Of the clinical features which predicted the genotype, peripheral neuropathy had the highest specificity (90%), negative predictive value (83%) and positive likelihood ratio (5.87) for the diagnosis of a nuclear DNA defect. The relatively low frequency of peripheral neuropathy among patients with PEO (21%), however, explains the low sensitivity (52%).

Both multinomial regression and decision tree analysis identified two other variables with predictive and classification ability: family history and hearing loss. Family history is clearly a useful feature in the differential diagnosis of mitochondrial disease. However, a negative family history in first-degree relatives could be explained by either a sporadic single mitochondrial DNA deletion or an autosomal recessive nuclear DNA defect. In the present sample, 50% of patients with a nuclear DNA defect did not have a family history of similar symptoms or phenotype. Hearing loss is a frequent feature in patients with m.3243A>G. In this study, in contrast, it was observed in only a small proportion of patients with a single mitochondrial DNA deletion or a nuclear DNA defect.

We believe that these findings have clinical implications. First, patients presenting with PEO and suspected mitochondrial disease should be carefully examined to exclude or confirm the presence of peripheral neuropathy, since this may be helpful in the selection of genetic tests. Second, the finding of peripheral neuropathy in a patient with PEO and a single mitochondrial DNA deletion should prompt the investigation to exclude alternative aetiologies. Third, certain combinations of clinical features are highly suggestive of the underlying genetic defect and may guide the diagnostic investigations. For instance, 90% of patients with PEO, peripheral neuropathy and no hearing loss, and 67% of patients with PEO, peripheral neuropathy and hearing loss, had a nuclear DNA defect or a point mutation of mitochondrial DNA (m.3243A>G), respectively. Genetic testing for these defects can be initially performed in DNA extracted from blood and, if confirmed, this may avoid the need for invasive procedures (Rahman and Hanna, 2009).

### Limitations

This study has several limitations. First, the design of the study did not allow a prospective standardized evaluation of patients. In this regard, the diagnosis of peripheral neuropathy was made on the basis of retrospectively collected clinical information and neurophysiological studies that were not always performed using the same protocol. All patients, however, had been assessed by experienced clinicians at the National Hospital for Neurology and Neurosurgery and a detailed and systematic collection of clinical information was carried out in all cases to minimize loss of data. Second, this was a single-centre study and the sample might not be representative of the whole patient population. However, the frequency distribution of genotypes among patients with PEO is similar to that observed in other case series (Holt *et al.*, 1989; Jackson *et al.*, 1995; Rodríguez-Hernández *et al.*, 2000), studies based on single-centre experience (Jiménez Caballero *et al.*, 2007), and population-based studies (Martikainen *et al.*, 2012). In addition, to minimize selection biases, all patients with PEO and mitochondrial disease that were assessed at our centre and with clinical information available were included in the study. Third, although this study comprised a large number of patients with mitochondrial disease, the sample size did not allow us to examine the predictive value of those clinical manifestations that were observed in only a minority of cases (e.g. stroke-like episodes, epilepsy, parkinsonism) or the predictive value of specific peripheral neuropathy subtypes (e.g. neuronopathy versus axonal with slowing of conduction velocity). Finally, this study only included patients with PEO due to mitochondrial disease. Therefore, results cannot be generalized to an unselected population of patients with ptosis or ophthalmoplegia but to patients with suspected mitochondrial disease in whom other disorders have been excluded on clinical grounds.

## Conclusion

This study highlights the phenotypic and genotypic heterogeneity of mitochondrial diseases but also that the analysis of a large case series may help establish consistent phenotype–genotype correlations. The results indicate that peripheral neuropathy is a rare finding in patients with single mitochondrial DNA deletions and that the presence of peripheral neuropathy is highly predictive of an underlying nuclear DNA defect, particularly *POLG* mutations. This observation will facilitate future development of more efficient diagnostic algorithms to aid clinicians when selecting and interpreting molecular genetic investigations.

## Funding

This study was supported by a Medical Research Council (MRC) Centre grant (G0601943), the UK NHS Specialised Service for Rare Mitochondrial Diseases of Adults and Children, and the National Institute for Health Research University College London (UCL) Hospitals/UCL Comprehensive Biomedical Research Centre. This work was undertaken at UCL Hospitals/UCL, which received a proportion of funding from the Department of Health's National Institute for Health Research Biomedical Research Centres funding scheme. MMR received grant funding from the National Institute of Neurological Disorders and Stroke/Office of Rare Diseases (1U54NS065712-01). MGH is also supported by the Myositis Support Group. AH received a research training fellowship from Caja Madrid Foundation, Spain.

## Supplementary material

Supplementary material is available at *Brain* online.

## Abbreviations

MELAS: mitochondrial encephalomyopathy, lactic acidosis and stroke-like episodes
PEO: progressive external ophthalmoplegia
PEO/CN: PEO with CNS involvement
SANDO: sensory ataxia, neuropathy, dysarthria and ophthalmoplegia
